# Dual‐scan conformal cone‐beam CT for targeted image‐quality improvement using dynamic collimation

**DOI:** 10.1002/acm2.70671

**Published:** 2026-06-23

**Authors:** Yuxiang Liu, Siqi Yuan, Xin Feng, Jianrong Dai

**Affiliations:** ^1^ National Cancer Center/National Clinical Research Center for Cancer/Cancer Hospital, Chinese Academy of Medical Sciences and Peking Union Medical College Beijing China

**Keywords:** adaptive radiotherapy, cone‐beam CT, conformal imaging, dual‐scan acquisition, dynamic collimation

## Abstract

**Background:**

Cone‐beam computed tomography (CBCT) is routinely used for image guidance in radiation therapy, but conventional full‐field CBCT acquisition may expose anatomically irrelevant regions and generate substantial scatter, which degrades soft‐tissue contrast and limits the accuracy of target localization and adaptive radiotherapy workflows. A clinically practical imaging strategy should improve image quality in the region of clinical interest while preserving sufficient full‐field anatomical information for patient setup and dose‐related assessment.

**Purpose:**

To improve image quality within clinically relevant regions without increasing the total imaging dose, this study developed a dual‐scan conformal cone‐beam CT method using dynamic collimation.

**Methods:**

A dual‐scan conformal acquisition strategy using dynamic collimation was designed. Dynamic collimation was defined as angle‐dependent shaping of the X‐ray field around a preplanned target region; it was implemented with a multi‐leaf collimator model in the digital phantom and patient‐data simulations and with the movable kV collimator in the physical phantom measurement. Collimator positions were calculated from forward projections of the target region, and a target‐region‐based intensity‐optimization model was used to allocate more photons to projection angles and detector regions contributing to the target region. The dual‐scan protocol, consisting of a low‐dose full‐field‐of‐view (FOV) scan and a higher‐dose conformal scan, was reconstructed using a regularized weighted least‐squares algorithm with anisotropic total‐variation regularization. The method was evaluated using a FORBILD head phantom, patient planning CT datasets, and an anthropomorphic head phantom measured on a Varian Edge on‐board imaging system.

**Results:**

The proposed method achieved the best overall image quality among the compared methods. In the FORBILD phantom, for target region 1 (VOI1), SSIM increased from 0.702 to 0.855, CNR increased from 0.514 to 1.463, and SNR1/SNR2 increased from 33.8/32.8 to 113.1/114.6 compared with conventional FDK reconstruction. In the prostate cancer case, CNR increased from 0.44 to 1.94, and SNR1/SNR2 increased from 25.6/23.3 to 74.6/82.4. In the measured anthropomorphic head phantom, CNR improved from 1.036 to 2.379, and SNR1/SNR2 improved from 42.8/50.1 to 89.8/98.0.

**Conclusion:**

The proposed dual‐scan conformal CBCT strategy using dynamic collimation improves local image quality in the clinically relevant target region while maintaining full‐FOV information through the two‐scan protocol. This method may provide a practical route toward patient‐specific CBCT guidance for adaptive radiotherapy.

## 1 INTRODUCTION

Cone‐beam computed tomography (CBCT) has become one of the most important tools for image‐guided radiation therapy (IGRT).^1^, ^2^ It is widely used to visualize patient anatomy before treatment and to support accurate patient setup. With the increasing clinical demand for adaptive radiotherapy (ART), more advanced applications of CBCT have also been explored. Daily anatomical information can also support online ART, in which a treatment plan is generated or adapted according to the anatomy of the day to maintain plan quality despite inter‐fraction variations.^3^, ^4^ However, CBCT acquisition introduces additional X‐ray exposure. Therefore, both the as‐low‐as‐reasonably‐achievable (ALARA) principle and the AAPM Task Group 75 report emphasize that imaging dose should not be considered negligible and that strategies to reduce imaging dose and the exposed anatomical volume should be pursued whenever possible, even when new acquisition and reconstruction techniques are required.^5^

Current clinical CBCT workflows still largely follow a relatively static paradigm. For different treatment sites, such as the head and neck, thorax, abdomen, and pelvis, a fixed set of scanning parameters is usually selected according to established clinical protocols. These parameters include tube potential or X‐ray spectrum, tube current, exposure time, collimator opening size, imaging field‐of‐view (FOV), and the use of a bowtie filter. Although this approach simplifies workflow and quality control, its limitations have become increasingly apparent in precision radiotherapy.^6^, ^7^ Patient anatomy varies substantially, even within the same treatment site; body size, tissue‐density distribution, and the relative positions of the target and organs at risk may differ greatly among individuals. Static scanning parameters therefore cannot be optimized for these patient‐specific differences and may result in either unnecessary imaging dose or insufficient image quality.^8^, ^9^ In addition, large‐field collimation inevitably irradiates anatomical regions that may be unrelated to the current treatment fraction or plan adaptation, which increases both patient dose and scatter‐induced noise in the projections. This can reduce soft‐tissue contrast, particularly around the tumor target and adjacent critical structures, and may compromise contouring accuracy.^10^, ^11^

Patient dose from CBCT imaging can be reduced by adjusting X‐ray fluence, beam energy, or the number of acquired projections. Most existing strategies focus on fluence reduction, commonly by lowering tube current uniformly or modulating it on a view‐by‐view basis according to patient thickness.^12^ Although these methods can reduce dose, they may also increase projection noise. Iterative reconstruction and projection‐denoising techniques have been investigated to control noise, and few‐view reconstruction has been explored to reduce the number of angular samples. However, these dose‐reduction approaches are usually not tailored to the specific imaging task. Volume‐of‐interest (VOI) CBCT can improve local image quality and reduce dose relative to full‐field CBCT by limiting the irradiated volume and reducing scatter generation within the patient. Previous studies have demonstrated that static copper collimation can reduce scatter substantially, improve the contrast‐to‐noise ratio (CNR), and reduce dose outside the target region.

Chen et al. performed VOI imaging of the breast using a copper plate with a circular aperture, and their results showed that this approach improved soft‐tissue contrast.^13^ Cho et al. systematically investigated the effectiveness of VOI CBCT in reducing scatter and radiation dose. Their experimental results demonstrated that, compared with full‐field CBCT, VOI CBCT reduced the imaging dose by 17%–26% while decreasing the scatter signal within the VOI by 85%–90%.^14^ Yang et al. applied the VOI imaging method to the imaging of intracranial stents and flow‐diverter devices, showing that this approach could obtain high‐quality diagnostic images while substantially reducing radiation exposure.^15^ Robar et al. implemented VOI CBCT imaging using a 2.35 MV beam from a linear accelerator, an electronic portal imaging device, and a multi‐leaf collimator (MLC), demonstrating that the image quality of this dynamic VOI imaging method was superior to that of full‐field imaging.^16^ Parsons et al. implemented VOI imaging on a Varian linear accelerator equipped with kV‐CBCT, achieving a 2.2‐fold improvement in CNR and a 15%–80% reduction in imaging dose.^17^, ^18^

This study proposes a patient‐specific dual‐scan conformal CBCT imaging method that adjusts dynamic collimation and tube current according to a preplanned target region and patient‐specific anatomy. The goal is to reduce unnecessary irradiation of noncritical regions while maintaining or improving image quality in clinically important regions, thereby optimizing image quality and radiation safety simultaneously.

The technical basis of this concept comes mainly from two validated imaging strategies. The first is automatic tube current modulation, which is widely used in diagnostic CT^19^, ^20^, ^21^, ^22^, ^23^ to adjust tube current according to patient size and angle‐dependent X‐ray attenuation, thereby reducing overall dose while maintaining image‐noise uniformity. The second is target‐ or volume‐of‐interest imaging in CT,^16^, ^17^, ^24^, ^25^, ^26^ which reduces scatter and imaging dose through physical collimation. The present study integrates and extends these strategies by proposing a dual‐scan conformal CBCT method using dynamic collimation.

The proposed conformal imaging method is analogous to rotational conformal radiotherapy.^27^, ^28^ In rotational conformal radiotherapy, the beam shape and intensity are adjusted at each incident angle so that the high‐dose region conforms to the tumor while sparing normal tissues. Similarly, during projection‐data acquisition, dynamic collimation and tube current modulation are used to adjust the field shape and intensity at each imaging angle. In the digital phantom and patient‐data simulations, dynamic collimation was implemented with an MLC model to approximate angle‐dependent field shaping. In the anthropomorphic phantom measurement, it was implemented with the movable kV collimator available on the Varian Edge OBI system. This rotational conformal imaging strategy can reduce imaging dose and scatter in non‐target regions and improve the utilization efficiency of imaging photons. In this study, digital phantoms and patient images were evaluated through simulation, and an anthropomorphic head phantom was tested experimentally to verify the effectiveness of the method.

## 2 MATERIALS AND METHODS

### 2.1 Dual‐scan conformal CBCT acquisition

**FIGURE 1:**
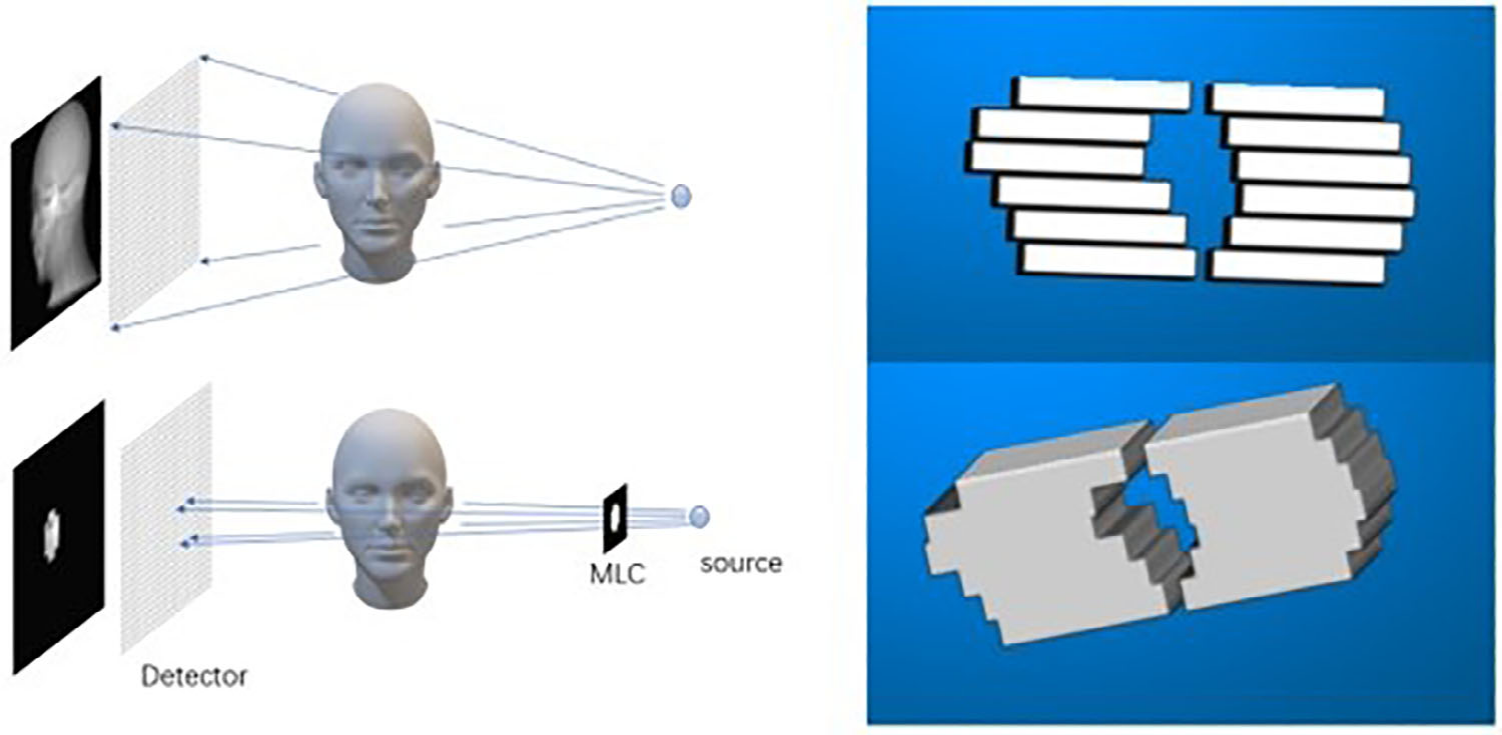
Schematic of conformal CBCT imaging with dynamic collimation (left) and CAD model of the MLC used in the simulation studies (right).

As shown in Figure 1, conformal scanning improves local image quality by reducing the irradiated imaging range around the target region. In this study, dynamic collimation was used as an umbrella term for angle‐dependent field shaping with available collimating hardware. For the digital phantom and patient‐data simulations, a high‐precision MLC model dynamically modulated the beam shape so that the X‐ray field conformed to the target region. For the physical phantom measurement, the same dynamic‐collimation concept was implemented using the movable rectangular kV collimator because the clinical OBI CBCT system was not equipped with an imaging MLC. Tube current modulation was then incorporated to adjust X‐ray intensity and preferentially allocate imaging dose to the target region.

### 2.2 Collimator position calculation for dynamic collimation

The calculation of collimator positions follows the principle used in conformal radiotherapy: the projected field is restricted to cover the target region. In the simulation studies, the collimating hardware was modeled as an MLC. First, the target region was delineated and converted into a binary image, with voxels inside the target region assigned a value of 1 and all other voxels assigned a value of 0. The binary image was then forward projected onto the detector plane, and the resulting projection was used to determine the MLC leaf positions. The projection image was binarized again; pixels with values greater than 1 were assigned 1, whereas all remaining pixels were assigned 0. For each leaf pair, the corresponding y‐direction range on the projection image was determined, and a logical operation was performed within that range to obtain the x‐direction coverage of the projected target region. Finally, the physical leaf positions were calculated from the source‐to‐MLC distance, source‐to‐detector distance, and projected leaf range.

**FIGURE 2:**
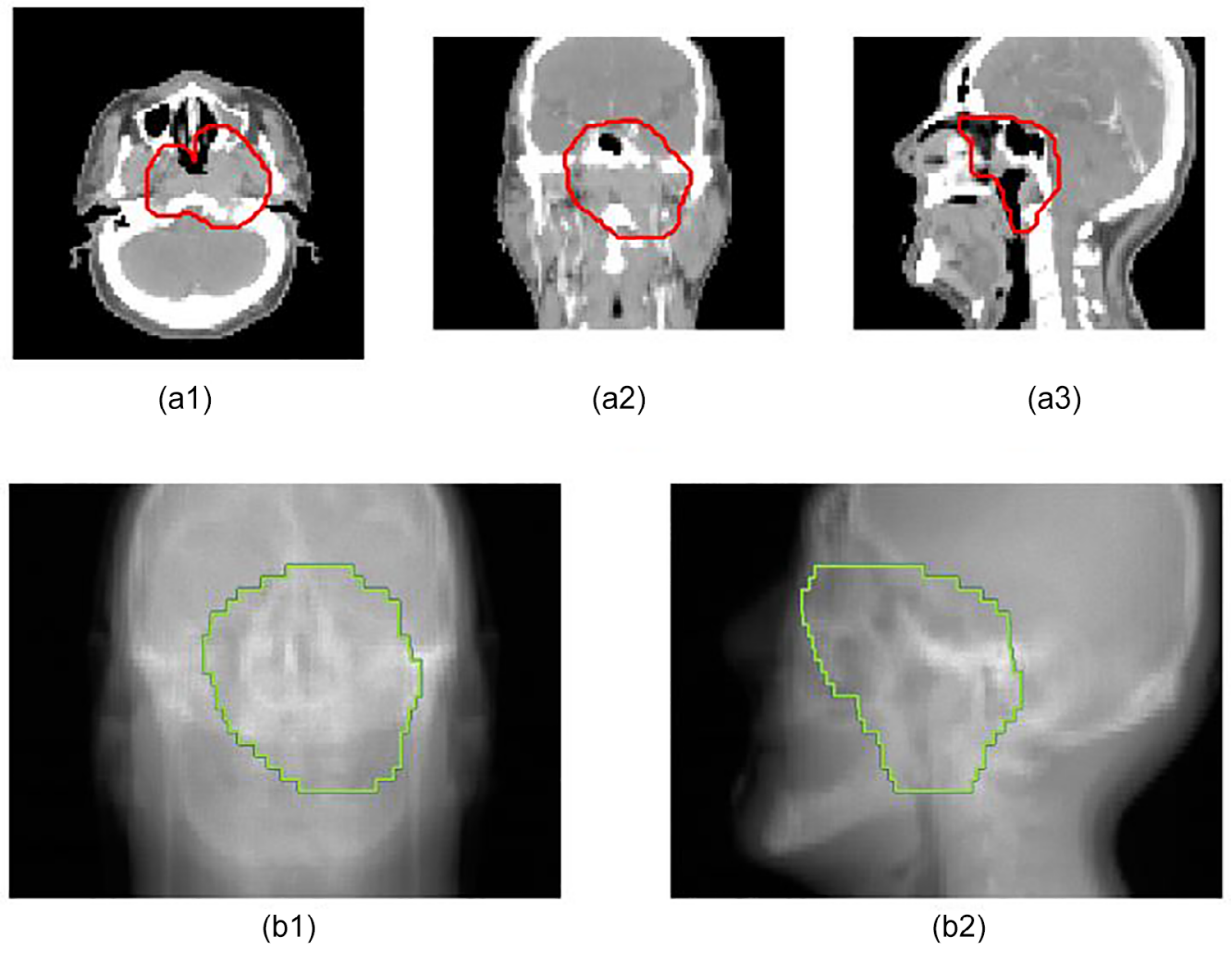
Example of MLC‐based dynamic collimation for an NPC patient. (a1–a3) Axial, coronal, and sagittal CT images of the patient with the corresponding VOI delineation. The red contours indicate the VOI used for conformal imaging. (b1, b2) Representative CT forward projections at different projection angles with the corresponding MLC‐defined aperture. The green contours indicate the projected shape of the MLC aperture on the detector plane.

Because the MLC leaf speed is limited, the number of control points can be reduced in practical implementation. If 720 projections are acquired uniformly over 360 degrees, the ideal control‐point spacing would be 0.5 degrees. In current accelerator‐based treatment planning, however, MLC control points are typically placed every 2–4 degrees. Therefore, when hardware constraints are present, fewer control points can be used. For each control point, the maximum x‐direction range of the binary projection within the corresponding angular interval is used to calculate the MLC leaf positions, ensuring full target‐region coverage from all directions while reducing irradiation of non‐target regions. Figure 2 shows an example for a nasopharyngeal carcinoma (NPC) patient, in which GTVnx expanded by 5 mm was selected as the target region. Its forward projection and the corresponding MLC leaf positions are shown in Figure 2b1,b2.

### 2.3 Intensity optimization

The proposed intensity‐optimization strategy was inspired by beam modulation in radiotherapy. Because CBCT image formation involves both projection acquisition and image reconstruction, incorporating the full reconstruction process into a rigorous optimization model would be computationally expensive. Therefore, a simplified model was adopted. Based on the positive relationship between photon fluence and image quality, the optimization objective was converted into maximizing the number of attenuated photons contributing to the target region while minimizing unnecessary attenuation in non‐target regions.

**FIGURE 3:**
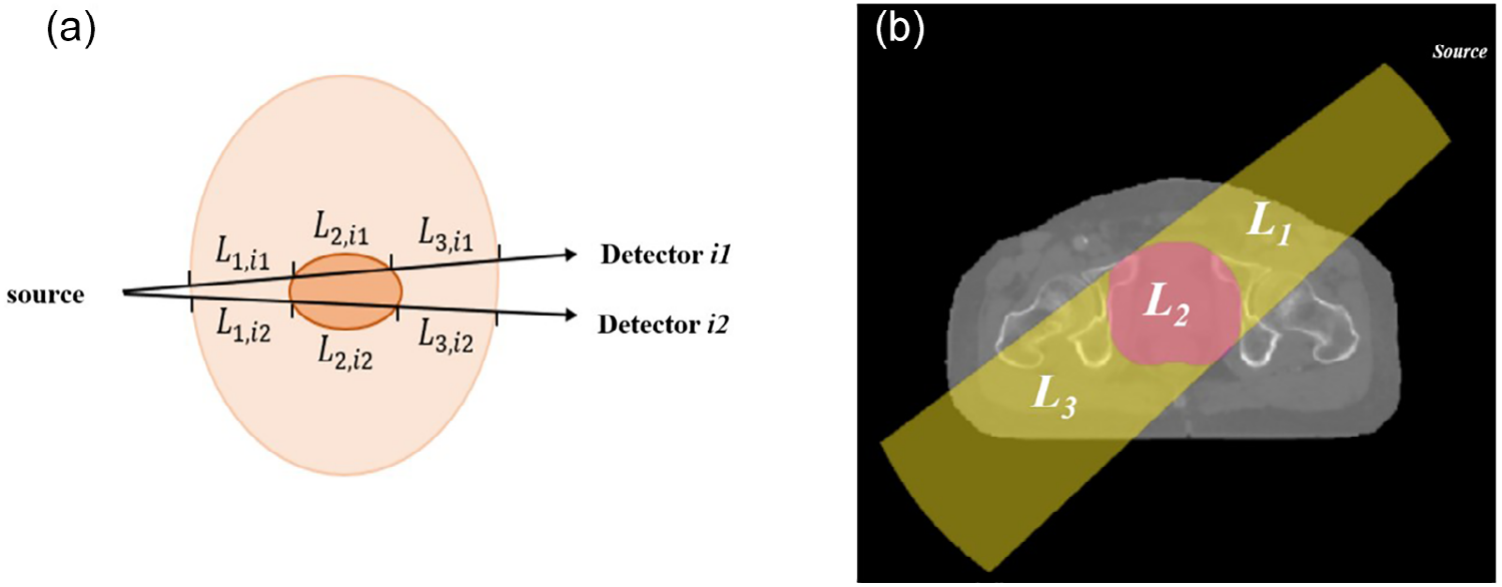
Illustration of beam paths used in the target‐region‐based intensity‐optimization model. (a) Schematic diagram showing the paths of individual X‐ray beams corresponding to different detector elements at the same projection angle. The darker central region represents the VOI. (b) Example patient image showing the regions traversed by the entire irradiation field, where L2 denotes the VOI and L1 and L3 denote non‐target regions before and after the beam passes through the VOI. The arrows indicate the beam direction, and i1 and i2 represent different detector elements.

As shown in Figure 3, L2 represents the target region, whereas L1 and L3 represent non‐target regions. According to the linear attenuation property of X‐rays, the numbers of photons attenuated inside and outside the target region at projection angle n can be expressed as follows:

(1) $$N_{n,inside}=N_{n,0}\sum_{i\subset I}e^{-\int_{L1,i}\mu dl\;}\left(1-\;e^{-\int_{L2,i}\mu dl\;}\right)$$

(2) $$\begin{array}{cc} N_{n,outside} & =N_{n,0}\sum_{i\subset I}\left[\left(1-\;e^{-\int_{L1,i}\mu dl\;}\right)\right.\\ & \left.\;+e^{-\int_{L1,i}\mu dl-\int_{L2}\mu dl\;}\left(1-\;e^{-\int_{L3,i}\mu dl\;}\right)\right] \end{array}$$

where n denotes the projection‐angle index, and i denotes the detector‐element index. I denotes the set of detector elements that are not blocked by the dynamic collimator at projection angle n. $N_{n,0}$ represents the number of source photons emitted toward a single detector element before attenuation at projection angle n. $N_{n,inside}$ and $N_{n,outside}$ represent the numbers of photons attenuated inside and outside the target region, respectively. L1,i and L3,i denote the non‐target path segments before and after the target region along the ray path, whereas L2,i denotes the path segment inside the target region. μ is the linear attenuation coefficient.

The equation can be simplified as follows:

(3) $$N_{n,\mathrm{inside}}=N_{n,0}$$

(4) $$N_{n,\mathrm{outside}}=B_{n}N_{n,0}$$

where $A_{n}$ is equal to $\sum_{i\subset I}e^{-\int_{L1,i}\mu dl\;}\left(1-\;e^{-\int_{L2,i}\mu dl\;}\right)$ and $B_{n}$ is equal to $\sum_{i\subset I}\left[\left(1-\;e^{-\int_{L1,i}\mu dl\;}\right)+e^{-\int_{L1,i}\mu dl-\int_{L2}\mu dl\;}\left(1-\;e^{-\int_{L3,i}\mu dl\;}\right)\right]$. They are angle‐dependent coefficients determined by the target and non‐target path lengths, attenuation distribution, and the unblocked detector‐element set at projection angle n.

To keep the imaging dose controlled during acquisition, the total number of emitted and detected photons was constrained to remain consistent across the compared protocols:

(5) $$\sum_{n=1}^{N}D_{n}N_{n,0}=N_{total}$$

where $D_{n}$ is the number of valid detector elements receiving signal under dynamic‐collimator shielding at projection angle n, and $N_{total}$ is the prescribed total photon budget used to constrain the compared acquisition protocols.

The optimization aims to increase the target‐region photon contribution while reducing unnecessary non‐target‐region attenuation. It determines the angular source fluence and, in the simulation studies, the MLC leaf positions. The objective function is:

(6) $$N_{n,0}=\arg\;\min_{N_{n,0}}\left(\alpha N_{total}-\sum_{i=1}^{n}N_{n,\mathrm{inside}}+\sum_{i=1}^{n}N_{n,\mathrm{outside}}\right)$$

where α is a dimensionless weighting coefficient used to balance the target‐region photon contribution and the non‐target‐region attenuation term. The optimization variable $N_{n,0}$ represents the incident photon number assigned to projection angle n.

### 2.4 Reconstruction algorithm

The iterative reconstruction method used in this study was modified from regularized weighted least squares (RWLS) with an anisotropic total‐variation (TV) regularization term. The RWLS objective function is expressed as follows:

(7) $$\begin{array}{cc} f & =\arg\min_{f}\left\{\Psi_{RWLS}\left(f\right)\right\}\\ & =\arg\;\min_{f}\left\{\frac{1}{2}{\left(Af-P\right)}^{T}W\left(Af-P\right)+\;\lambda_{RWLS}R\left(f\right)\right\} \end{array}$$

In this expression, f denotes the reconstructed image vector, $\Psi_{RWLS}\left(f\right)$ denotes the RWLS objective function, A is the system matrix that maps the image to the projection domain, P is the measured projection vector after logarithmic transformation, and Af−P is the projection residual. W is a diagonal weighting matrix used to weight different projection pixels according to their noise level, $\;\lambda_{RWLS}$ is the regularization parameter controlling the strength of the image‐prior constraint, and R(f) denotes the regularization term, which is implemented with anisotropic TV regularization in this study. The gradient and Hessian surrogate of the objective function are expressed as follows:

(8) $$\Psi_{RWLS}^{\prime }\;\left(f\right)=A^{T}W\left(Af-P\right)+\;\lambda_{RWLS}R{\left(f\right)}^{\prime }$$

(9)

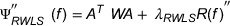

where $A^{T}$ denotes the transpose of the system matrix, corresponding to the back‐projection operator. $\Psi_{RWLS}^{\prime }\left(f\right)$ and 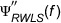 denote the gradient and Hessian surrogate of the RWLS objective function, respectively. $R{\left(f\right)}^{\prime }$ and 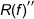 denote the first derivative and the surrogate second‐order curvature of the regularization term. The RWLS objective function was solved iteratively using a preconditioned conjugate‐gradient algorithm, and the separable quadratic surrogate method^29^, ^30^ was used to accelerate convergence.

RWLS reduces the noise level of reconstructed images and eliminates streak artifacts commonly seen in low‐dose scanning by establishing a physically realistic statistical model and introducing image‐prior constraints. In conventional CBCT scanning, the weighting matrix is calculated from the measured projection intensity, which approximately corresponds to the inverse of the variance of the measured data. Thus, for high‐noise regions, the algorithm automatically assigns smaller weights to reduce their influence on the reconstruction results; for low‐noise regions (high signal intensity), larger weights are assigned, thereby achieving a statistically optimal maximum‐likelihood estimate. In conformal reconstruction, the weighting matrix W is adjusted according to the different intensity distributions as follows:

(10) $$W=B\left(u,v\right)N_{n,0}\left(u,v\right)\;exp\left(-P\left(u,v\right)\right)$$

where, u and v denote the detector‐pixel coordinates, B(u,v) represents the bowtie‐filter or fluence‐shaping factor on the intensity distribution, $N_{n,0}\left(u,v\right)$ denotes the initial beam intensity distribution for detector pixel (u, v) after the two scans are combined, and (u,v) denotes the measured logarithmic projection value. Therefore, exp(−P(u,v)) represents the estimated transmission signal used to construct the statistical weight.

This study used the anisotropic TV regularization function provided by LEAP.^31^, ^32^ This function $R_{\mathrm{TV}}\left(f\right)$ is based on a Huber‐type loss, as shown below:

(11) $$R_{\mathrm{TV}}\;\left(f\right)=\beta \sum_{j}\sum_{i\in N\left(j\right)}\rho_{\delta }\left(f_{j}-f_{i}\right)$$

where β is the regularization‐strength parameter controlling the overall strength of the TV penalty, j denotes the index of the current voxel, N(j) denotes the set of neighboring voxels of voxel j, and i∈N(j) denotes a neighboring voxel included in this set. $f_{j}$ and $f_{i}$ are the attenuation‐coefficient values of voxels j and i, respectively, and $\rho_{\delta }\left(.\right)$ is the Huber loss function applied to the local image difference $f_{j}-f_{i}$. The Huber loss function is defined as follows:

(12) $$\rho_{\delta }\;\left(x\right)=\left\{\begin{array}{c} \frac{1}{2}x^{2}\;,\;\;\left|x\right|<\delta \;\\ \delta \left|x\right|-\frac{1}{2}\delta^{2}\;,\;\left|x\right|\geq \delta \end{array}\right.$$

where x denotes the local image difference, and δ is the Huber threshold parameter that controls the transition between the quadratic and linear regions. This parameter should be selected based on the expected difference in attenuation coefficients between the materials to be distinguished.

According to the characteristics of rotational conformal imaging, the TV regularization function was divided into two parts:

(13) $$R\;\left(f\right)=\;\lambda_{1}\;R_{1}\left(\;f_{out-VOI}\right)+\lambda_{2}\;R_{2}\left(\;f_{in-VOI}\right)$$

where the first term denotes the image outside the target region and the second term denotes the image inside the target region. The two regularization terms correspond to the outside and inside of the target region, respectively. Because the region outside the target region is affected by more severe noise and scatter, the outside‐region regularization strength should be greater than the inside‐region strength.

### 2.5 Evaluation metrics and test data

To comprehensively evaluate the performance of the proposed method, the relative root mean square error (RRMSE), structural similarity index measure (SSIM), CNR, and signal‐to‐noise ratio (SNR) were used as quantitative evaluation metrics.

For the digital phantom experiments, the FORBILD head phantom was used for comparative testing. To evaluate the performance of the proposed method at different spatial locations, two spherical regions with different positions were predefined in the phantom. Each sphere had a radius of 30 mm and was used as an independent target region (VOI1 and VOI2) for conformal scanning and reconstruction. During simulation, dynamic collimation was implemented with an MLC model with a leaf thickness of 5 mm at the isocenter plane.

For the patient‐data experiments, two typical radiotherapy planning CT datasets were selected as reference images for simulation: (1) a NPC case, in which the target region was generated by uniformly expanding the primary nasopharyngeal gross tumor volume by 5 mm in three dimensions (GTVnx + 5 mm); and (2) a prostate cancer case, in which the target region was generated by uniformly expanding the prostate clinical target volume by 5 mm in three dimensions (CTV + 5 mm). These expansion strategies are consistent with routine clinical target‐delineation practice and were intended to ensure adequate image quality at and around the target‐region boundary.

The experiments were conducted using the simulation platform developed for this study. In the simulations, the CBCT imaging‐system parameters were as follows: SAD was 1000 mm and SDD was 1500 mm; the projection angle covered 360 degrees, with 720 projections for the head‐and‐neck case and 900 projections for the pelvic case; the flat‐panel detector size was 1024 × 768 pixels, with a pixel size of 0.388mm^2^; and the reconstruction voxel size was 1 mm^3^. For the pelvic case, the detector was shifted by 160 mm to expand the imaging range. In conventional CBCT, the exposure was set to an average of 0.2 mAs per projection for the head‐and‐neck case and 1.0 mAs per projection for the pelvic case. In the two‐scan protocol, the first full‐field scan was set to an average of 0.1 mAs per projection for the head‐and‐neck case and 0.6 mAs per projection for the pelvic case, whereas the higher‐dose conformal scan was set to an average of 0.4 mAs per projection for the head‐and‐neck case and 1.5 mAs per projection for the pelvic case. Based on these settings, the intensity of each projection was adjusted while the total number of imaging photons was kept unchanged.

**FIGURE 4:**
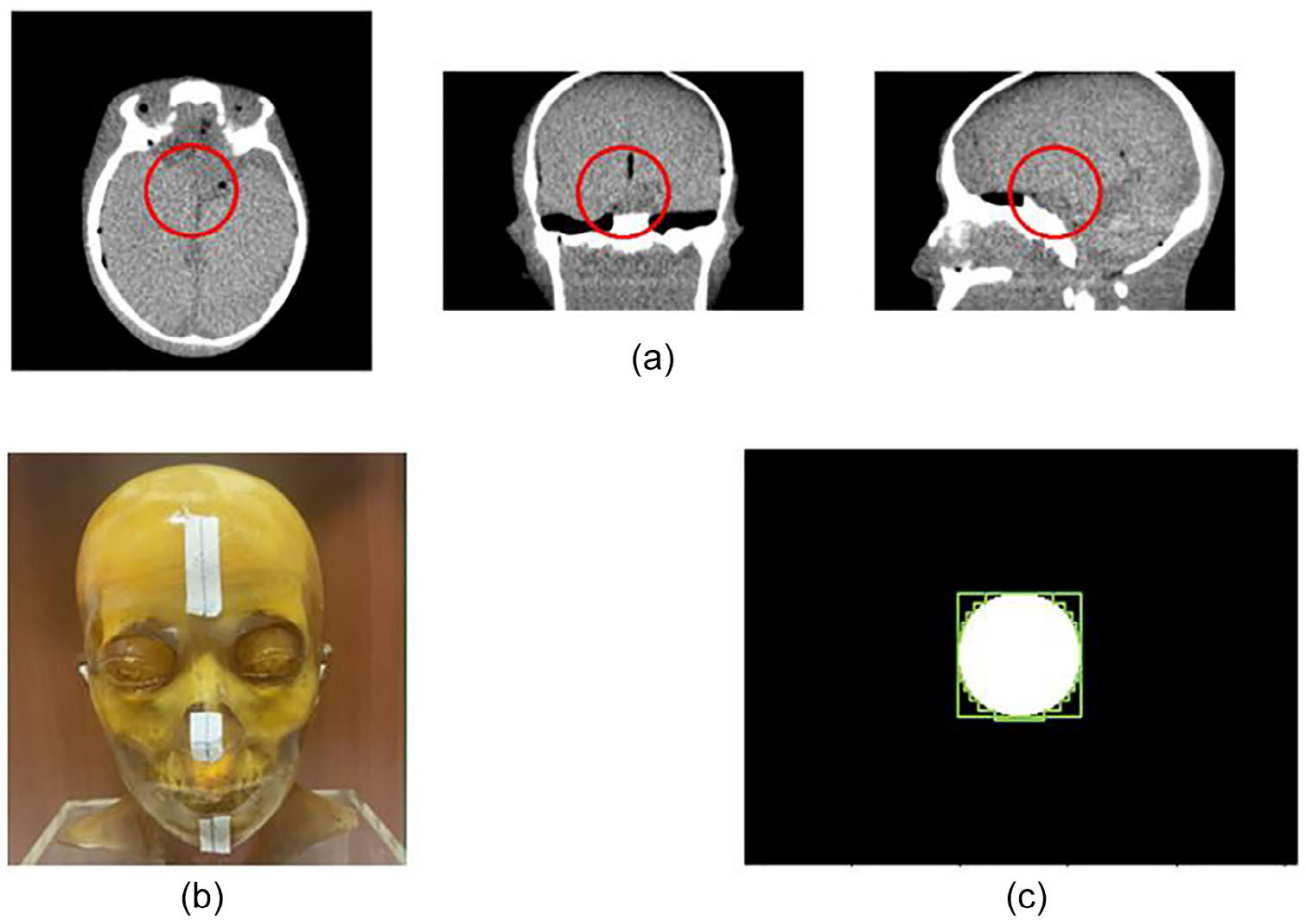
Target region and conformity schematic for the anthropomorphic head phantom: (a) CBCT image of the phantom and the corresponding VOI; (b) physical phantom; (c) schematic of collimator‐based conformity.

For the measurement experiment, an anthropomorphic head phantom (Figure 4b) was used, and a spherical region with a radius of 30 mm inside the phantom was selected as the target region (Figure 4a). The experiment was performed on the OBI CBCT imaging system of a Varian Edge linear accelerator. Because the kV‐CBCT system was not equipped with an imaging MLC, dynamic collimation for the measured experiment was implemented using the movable collimator provided with the system, which consists of two leaf pairs in the X and Y directions. Figure 4c compares the conformity that would be achieved by an MLC with a 5‐mm leaf thickness at the isocenter plane with the rectangular conformity achieved by the movable collimator on the existing kV‐CBCT system.

### 2.6 Method comparison

In the method‐comparison experiment, the proposed reconstruction algorithm was compared with the conventional FDK algorithm, and the proposed target‐region‐based intensity‐optimization strategy was compared with common attenuation‐based automatic tube current modulation methods,^18^, ^23^, ^33^ which are based on the automatic tube current modulation theory proposed by Gies et al.^19^, ^20^ In this theory, projection noise decreases as the number of acquired photons increases; therefore, tube current is adjusted by selecting an appropriate number of photons at each angle to minimize the total noise:

(14) $$\frac{\partial \sigma^{2}}{\partial N_{n,0}}=0$$

where $\sigma^{2}$ denotes the total projection‐noise variance to be minimized, and N_i,0 denotes the incident source photon number, or tube‐current level, assigned to projection angle n before attenuation. Setting the derivative of $\sigma^{2}$ with respect to $N_{n,0}$ to zero gives the noise‐based optimality condition for attenuation‐based tube current modulation. In the implementation, the attenuation at each projection angle was calculated using the actual beam‐shape approximation method proposed by Son et al.^33^ This method accounts for the actual X‐ray beam shape on the detector in CBCT. Representative paths covering the irradiated beam range are selected, and the average attenuation along these paths is calculated to more accurately reflect the overall attenuation characteristics of X‐rays passing through the object. This avoids potential bias caused by a single central‐ray approximation. Finally, the beam intensity required at each projection angle is expressed as follows:

(15) $$N_{n,0}=\frac{N_{total}}{\sum_{n=1}^{N}\sqrt{C_{n}}}\cdot \sqrt{C_{n}}$$

where $N_{n,0}$ denotes the incident source photon number assigned to projection angle n, $N_{total}$ denotes the prescribed total photon budget, and N is the total number of projection angles. $C_{n}=e^{\int \mu dl}$ is the attenuation‐compensation coefficient for projection angle n, calculated from the line integral of the attenuation coefficient μ along the representative beam paths; μ is the linear attenuation coefficient and dl is the differential path length. The denominator normalizes the angular fluence distribution so that the sum of photons over all projection angles remains equal to $N_{total}$. The intuitive interpretation of this algorithm is that projection angles with greater attenuation require higher tube current. The modulated beam intensity is proportional to the square root of the attenuation at the corresponding projection angle. Therefore, X‐ray paths with longer penetration lengths or greater attenuation use increased tube current, whereas angles with lower attenuation use reduced tube current, improving the allocation of imaging photons.

Six acquisition and reconstruction schemes were compared, as summarized in Table S1: (1) FDK‐s1: one full‐field scan with constant beam intensity at all angles, followed by FDK reconstruction; (2) FDK‐TCM‐s1: one full‐field scan with angular intensity modulation based on attenuation‐based TCM, followed by FDK reconstruction; (3) IT‐s1: one full‐field scan with constant beam intensity, followed by RWLS reconstruction; (4) FDK‐TCM‐s2: one low‐dose full‐field scan and one conformal scan with attenuation‐based TCM, followed by FDK reconstruction; (5) IT‐TCM‐s2: one low‐dose full‐field scan and one conformal scan with attenuation‐based TCM, followed by RWLS reconstruction; and (6) Proposed: one low‐dose full‐field scan and one conformal scan with the proposed target‐region‐based intensity‐optimization strategy, followed by RWLS reconstruction.

## 3 RESULTS

### 3.1 Digital phantom test results

**FIGURE 5:**
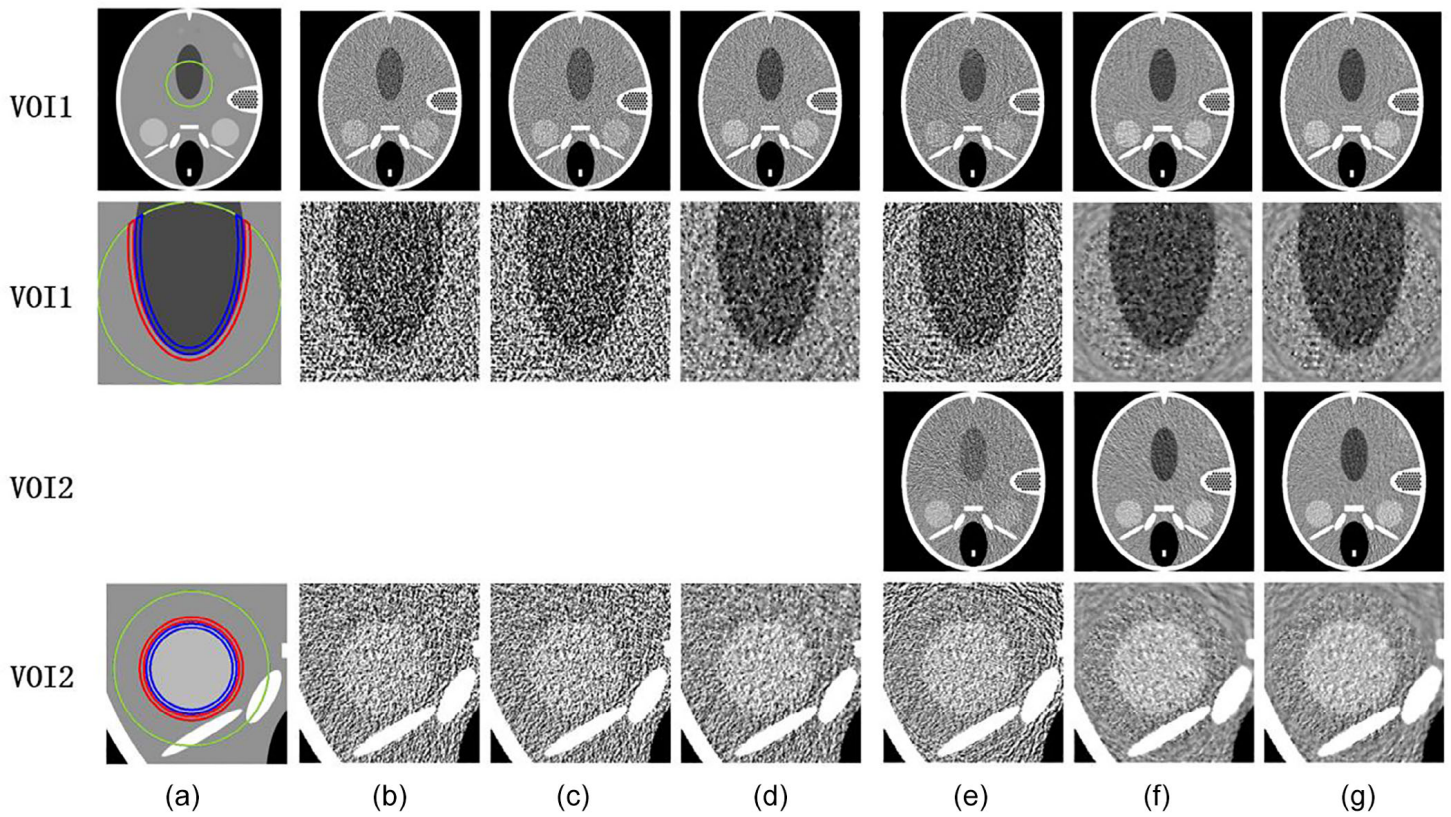
Digital phantom results. Simulation results of the FORBILD head phantom with two target regions and different imaging methods (a–g): reference image and target region, FDK‐s1, FDK‐TCM‐s1, IT‐s1, FDK‐TCM‐s2, IT‐TCM‐s2, and Proposed.

In the comparative experiment using the FORBILD head phantom, two spherical target regions with different spatial locations (both with a radius of 30 mm) were systematically tested. As shown in Figure 5, the first and second rows show the imaging results for VOI1, whereas the third and fourth rows show the imaging results for VOI2. Visual comparison shows that conventional FDK reconstruction (FDK‐s1) produced images with relatively high noise and reduced tissue‐boundary clarity. In contrast, the proposed dual‐scan conformal imaging method produced lower noise and clearer anatomical details within the target region.

Table 1 summarizes the quantitative evaluation results for VOI1 and VOI2. Taking VOI1 as an example, the proposed method achieved the best performance across all metrics: SSIM reached 0.855, representing a 21.8% improvement over FDK‐s1 (0.702); CNR reached 1.463, which was 2.85 times that of FDK‐s1 (0.514); and SNR1 and SNR2 reached 113.084 and 114.592, representing increases of 234.4% and 248.9%, respectively. These results demonstrate the ability of the proposed dual‐scan conformal imaging method to improve target‐region image quality. Although IT‐TCM‐s2 also used conformal scanning and iterative reconstruction, its performance remained slightly inferior to that of the proposed method, indicating that the proposed intensity‐optimization strategy provides more efficient dose allocation than conventional attenuation‐based TCM.

**TABLE 1:** Quantitative results for the two target regions in the FORBILD head phantom.

Target region	Method	SSIM	RRMSE	CNR	SNR1	SNR2
VOI1	FDK‐s1	0.702	0.396	0.514	33.839	32.834
	FDK‐TCM‐s1	0.704	0.396	0.516	33.978	32.978
	IT‐s1	0.825	0.396	0.944	67.860	69.550
	FDK‐TCM‐s2	0.766	0.396	0.671	46.562	44.758
	IT‐TCM‐s2	0.850	0.395	1.343	101.092	102.314
	Proposed	0.855	0.394	1.463	113.084	114.592
VOI2	FDK‐s1	0.745	0.385	0.278	36.149	38.905
	FDK‐TCM‐s1	0.748	0.385	0.284	36.763	39.511
	IT‐s1	0.813	0.385	0.410	53.410	61.181
	FDK‐TCM‐s2	0.802	0.385	0.374	48.428	52.332
	IT‐TCM‐s2	0.853	0.383	0.736	94.983	108.794
	Proposed	0.854	0.383	0.744	95.328	111.176

*Note*: SNR1 and SNR2 were calculated using the regions outlined in Figure 5a, and CNR was calculated using the two regions.

**FIGURE 6:**
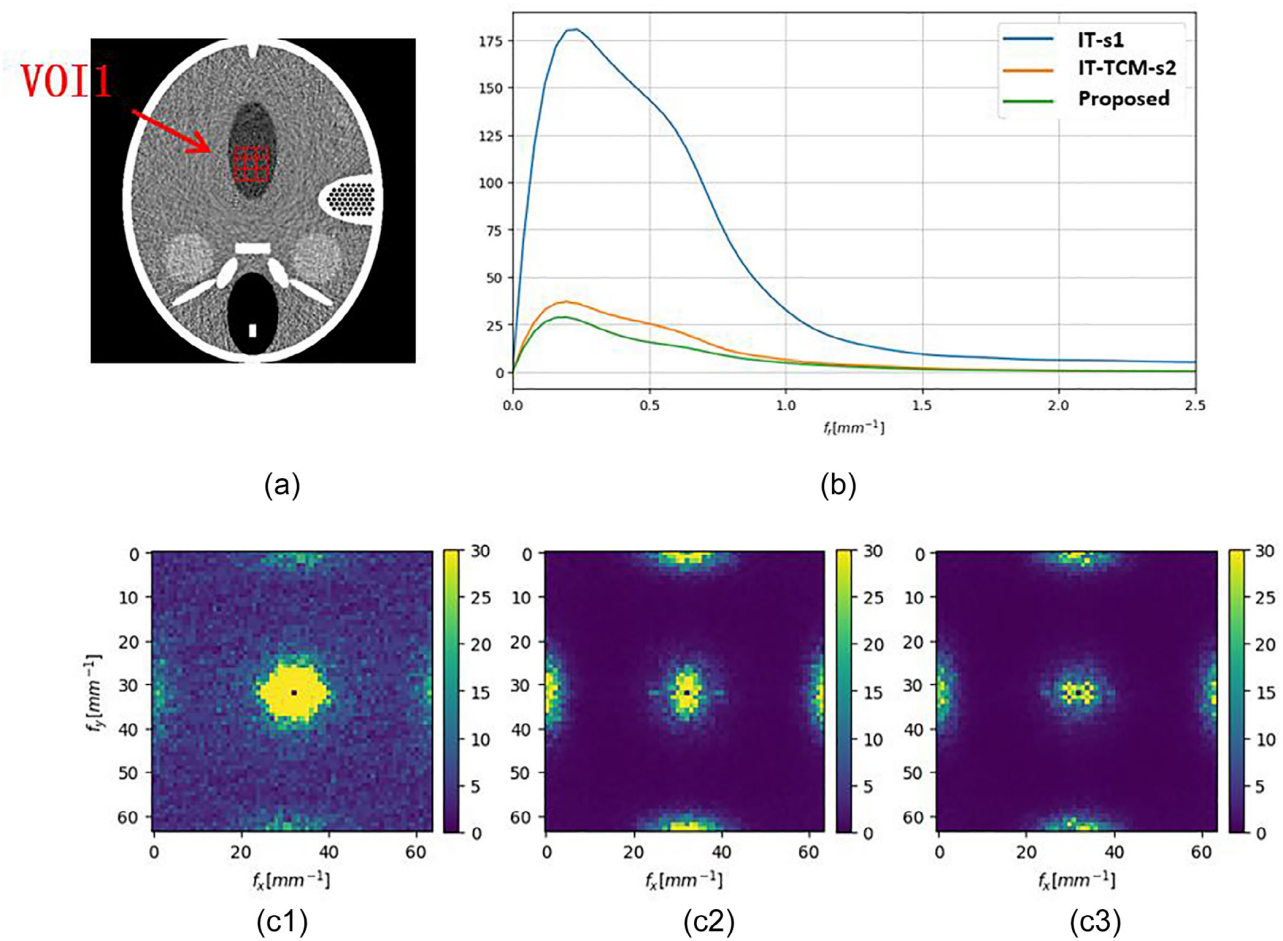
(a) Region used for NPS calculation and 1D NPS curves in (b). (c) 2D NPS distributions for IT‐s1(c1), IT‐TCM‐s2(c2), and proposed(c3).

To further analyze the noise characteristics of different methods, NPS analysis was performed. As shown in Figure 6a, the red boxes indicate the uniform regions selected for NPS calculation, and nine uniform regions were selected for noise‐distribution statistics. The 1D NPS curves in Figure 6b compare the noise characteristics of IT‐s1, IT‐TCM‐s2, and the proposed method. IT‐s1 showed the highest noise level and the largest curve peak, indicating substantial residual noise when conventional single scanning was combined with iterative reconstruction. IT‐TCM‐s2 showed an intermediate noise level, indicating that existing TCM provides some noise suppression. The proposed method produced a substantially lower noise curve, especially in the low‐frequency range, suggesting effective suppression of low‐frequency noise components.

The 2D NPS distributions in Figure 6c present the spatial characteristics of noise. The noise region in C1 (IT‐s1) was the largest and most concentrated, indicating a spatially nonuniform noise distribution. The noise region in C2 (IT‐TCM‐s2) was reduced but still showed obvious concentration. In C3 (Proposed), the noise region was the smallest and more uniformly distributed. These results indicate that the proposed intensity‐optimization method not only reduces the overall noise level but also improves spatial noise uniformity, which is important for accurate identification of lesion boundaries.

### 3.2 Patient data test results

**FIGURE 7:**
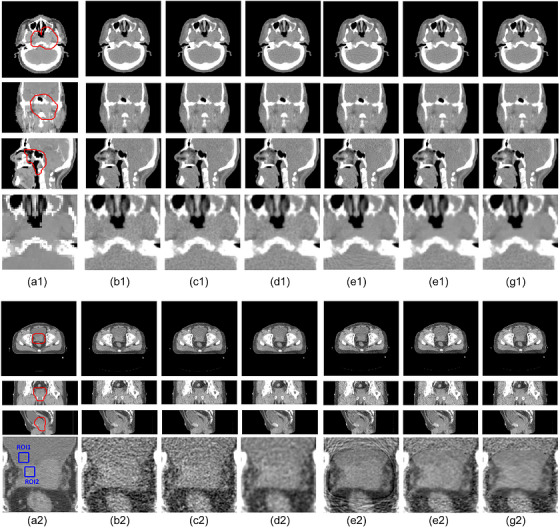
Patient simulation results. (a1–g1) Simulated results for the NPC patient with different imaging methods. (a2–g2) Simulated results for the prostate cancer patient with different imaging methods. The compared methods include reference image and target region (a1/a2), FDK‐s1(b1/b2), FDK‐TCM‐s1(c1/c2), IT‐s1(d1/d2), FDK‐TCM‐s2(e1/e2), IT‐TCM‐s2(f1/f2), and proposed(g1/g2).

This part of the study focused on full‐FOV imaging methods. As shown in Figure 7a1–g1, the first, second, and third rows of the simulated results for the NPC patient show axial, coronal, and sagittal images, respectively, and the fourth row shows enlarged target‐region images from the first row. The proposed method exhibited the best image quality in the target region (the primary nasopharyngeal tumor target expanded by 5 mm): the tumor boundary was clearer, surrounding soft‐tissue contrast was improved, and the noise level was substantially reduced. By contrast, FDK‐s1 produced images with higher noise and less distinct tumor boundaries. FDK‐TCM‐s1 /IT‐s1 improved image quality to some extent through TCM, but the improvement was limited. FDK‐TCM‐s2/IT‐TCM‐s2 further improved image quality, but detail preservation was still slightly inferior to that of the proposed method.

Figure 7a2–g2 shows the simulated results for the prostate cancer patient. The first row shows axial images, the second row shows sagittal images, the third row shows coronal images, and the fourth row shows enlarged target‐region images from the first row. Because the pelvic region has large tissue‐density differences and a wider imaging range, conventional CBCT faces stronger scatter challenges. The proposed method showed excellent image quality within the target region (the prostate clinical target expanded by 5 mm) and more clearly distinguished the boundary between the prostate and surrounding tissues. The improvement was more pronounced in the prostate cancer case than in the NPC case, mainly because scatter is more severe in the pelvis and the advantages of conformal imaging in scatter suppression were more fully exploited.

Table S2 summarizes the SSIM and RRMSE results for the NPC and prostate cancer patient simulations. For the NPC patient, the proposed method achieved the best SSIM (0.846) and RRMSE (0.424). For the prostate cancer patient, the proposed method achieved the highest SSIM (0.761), whereas the RRMSE was comparable to the best‐performing method. The overall metrics for the prostate case were slightly lower than those for the NPC case, reflecting the greater difficulty of pelvic imaging due to a larger imaging range, more complex tissue‐density distribution, and higher scatter level. Nevertheless, the proposed method showed clear image‐quality advantages in the target region, further supporting its applicability in complex clinical scenarios.

Figure S1A shows the ROIs selected for CNR and SNR calculation in the prostate cancer patient, with ROI1 and ROI2 located at different positions inside the target region. Table 2 lists the quantitative evaluation results for each method. The proposed method achieved a CNR of 1.944, which was 4.39 times that of FDK‐s1 (0.443). SNR1 and SNR2 reached 74.615 and 82.434, representing increases of 191.7% and 253.9%, respectively, compared with FDK‐s1. These improvements indicate that the proposed method can effectively improve target‐region image quality and provide more reliable image guidance for precision radiotherapy.

**TABLE 2:** CNR and SNR results for the prostate cancer patient and anthropomorphic head phantom measurement.

Dataset	Method	CNR	SNR1	SNR2
Prostate cancer patient	FDK‐s1	0.443	25.584	23.295
	FDK‐TCM‐s1	0.663	27.585	32.535
	IT‐s1	1.298	55.241	67.775
	FDK‐TCM‐s2	1.833	63.560	73.892
	IT‐TCM‐s2	1.663	64.917	68.733
	Proposed	1.944	74.615	82.434
Phantom	FDK‐s1	1.036	42.766	50.079
	FDK‐TCM‐s1	1.170	44.230	56.176
	IT‐s1	1.790	74.903	80.384
	FDK‐TCM‐s2	2.057	84.210	87.769
	IT‐TCM‐s2	2.224	83.987	84.070
	Proposed	2.379	89.841	97.993

### 3.3 Anthropomorphic head phantom measurement results

**FIGURE 8:**
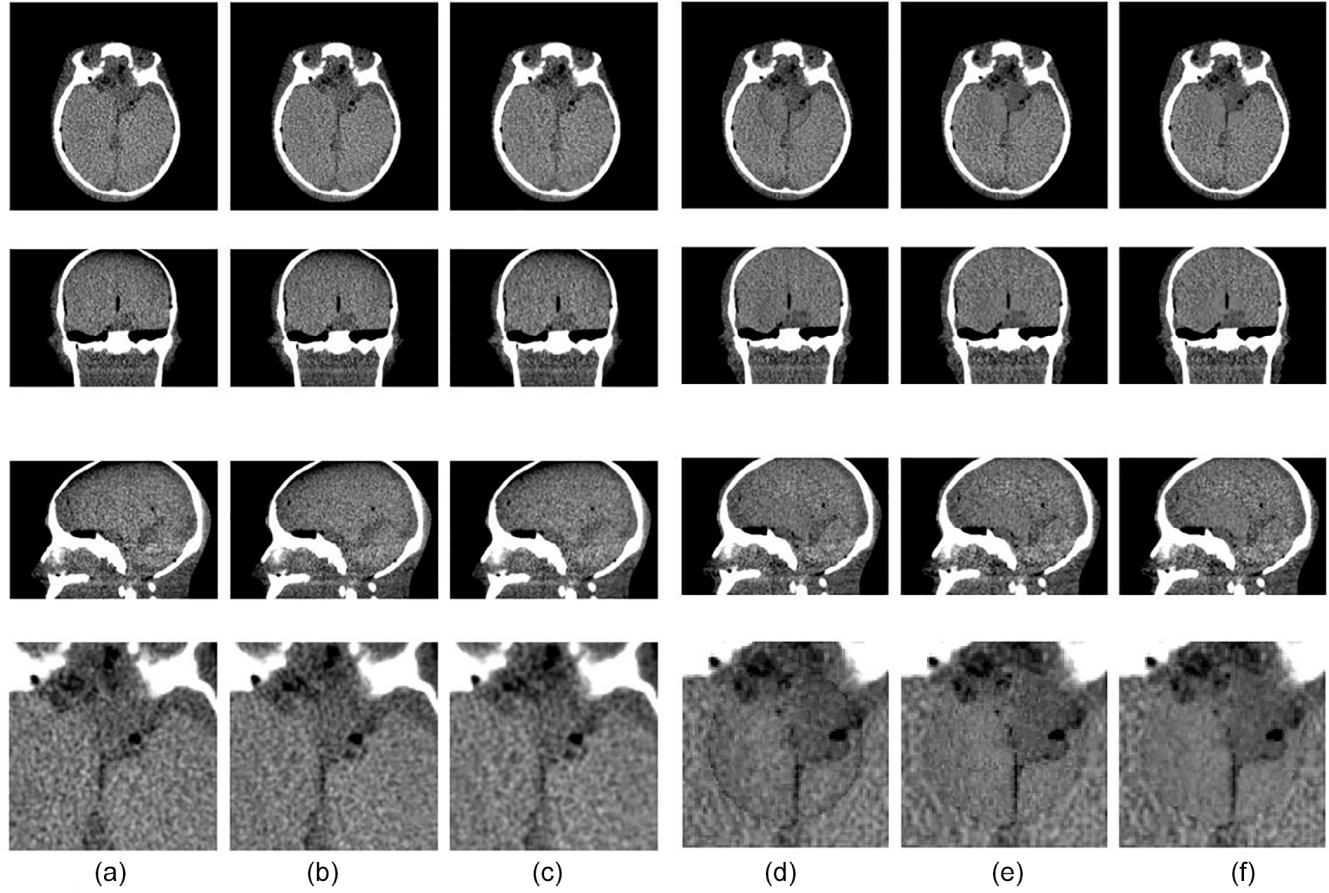
Experimental results for the anthropomorphic head phantom of different imaging methods: FDK‐s1(a), FDK‐TCM‐s1(b), IT‐s1(c), FDK‐TCM‐s2(d), IT‐TCM‐s2(e), and proposed(f).

Figure 8 shows the measured results for the anthropomorphic head phantom. The first row shows axial images, the second row shows coronal images, the third row shows sagittal images, and the fourth row shows enlarged target‐region images from the first row. Visual comparison shows that the proposed method achieved the best image quality within the target region: anatomical boundaries were clearer, noise was markedly reduced, and the overall image showed higher contrast and better uniformity. By contrast, FDK‐s1 showed obvious image noise, which obscured fine details. FDK‐TCM‐s1 and FDK‐TCM‐s2 provided limited improvements, and IT‐TCM‐s2 improved image quality but still did not perform as well as the proposed method. These observations are consistent with the simulation results and verify the effectiveness of the proposed method under actual hardware conditions.

Figure S1B shows the ROIs selected for CNR and SNR calculation in the anthropomorphic head phantom, with ROI1 and ROI2 located at different positions inside the target region. Table 2 lists the quantitative evaluation results for each method in detail. The proposed method achieved a CNR of 2.379, representing a 129.6% improvement over FDK‐s1. SNR1 and SNR2 reached 89.841 and 97.993, representing improvements of 110.1% and 95.7%, respectively, compared with FDK‐s1.

## 4 DISCUSSION

This study systematically investigated a dual‐scan conformal CBCT imaging method that integrates dynamic collimation, target‐region‐based intensity optimization, and regionally weighted iterative reconstruction into a patient‐specific precision imaging strategy. Compared with conventional static‐parameter imaging, the proposed method dynamically adjusts the field shape and intensity at each imaging angle according to patient anatomy and clinical requirements, thereby improving image quality within the target region.

In terms of scatter suppression, dynamic collimation reduces the irradiated volume and consequently decreases scatter contamination in the projections. Digital phantom experiments showed that reducing the imaging range around the target region decreased the scatter level substantially. In addition, the proposed intensity‐optimization algorithm maximizes the number of attenuated photons contributing to the target region while constraining unnecessary dose outside the target region. Unlike traditional TCM algorithms based mainly on noise‐uniformity assumptions, the proposed model directly reflects the technical characteristics of conformal imaging. Experimental comparisons showed that the proposed method outperformed conventional TCM across multiple evaluation metrics and achieved superior low‐frequency noise suppression and more uniform spatial noise distribution in the NPS analysis.

The proposed dual‐scan full‐FOV method is more consistent with clinical requirements in radiotherapy. In IGRT and ART applications, clinicians require high image quality in the target region but also need whole‐patient images for setup verification and dose calculation. By combining one low‐dose full‐field scan with one higher‐dose conformal scan, the method improves target‐region image quality while maintaining full‐FOV information. In addition, the regionally weighted TV‐regularized iterative reconstruction strategy accounts for the different noise characteristics inside and outside the target region. Stronger regularization outside the target region suppresses noise and artifacts, whereas moderate regularization inside the target region reduces noise while preserving image details. The experimental results showed that, without increasing the total imaging dose, the method substantially improved SNR and CNR in the target region.

The relatively pronounced improvement observed in the measurement experiment may be related to two factors. First, the actual CBCT system contains nonideal factors, such as detector‐response nonlinearity, source‐output fluctuation, and mechanical uncertainties, whereas the simulation model represents a more idealized acquisition process. The proposed method may be more robust to these nonideal effects because of optimized photon allocation and iterative reconstruction. Second, the scatter distribution in measured data differs from that in the simplified simulation model. Therefore, the physical phantom results provide additional evidence that dynamic collimation and target‐region‐based intensity optimization can improve target‐region image quality under realistic acquisition conditions.

Previous studies have also explored dynamic collimation or treatment‐beam‐based imaging. Parsons et al.^18^ used a dynamic collimator combined with tube current modulation to acquire projections, followed by extrapolation, interpolation, and FDK reconstruction within the region of interest. Poludniowski et al.^34^ proposed CT reconstruction from portal images acquired during volumetric‐modulated arc therapy (VMAT), termed VMAT‐CT, and developed an improved FBP algorithm for truncated projections. Zhao et al.^35^, ^36^, ^37^ further improved VMAT‐CT image quality and expanded its applications. These approaches dynamically adjust the field shape and intensity at each imaging angle. In contrast, the present study introduces a target‐region‐based intensity‐optimization algorithm designed for CBCT image guidance, rather than relying solely on attenuation‐based TCM or treatment‐prescription‐based intensity modulation.

In this study, the total emitted photon budget was constrained to be comparable among the evaluated protocols. Therefore, the observed image‐quality improvement was mainly attributed to spatial redistribution of imaging fluence and reduced scatter rather than to an increase in the total photon budget. However, the local dose distribution is not identical to that of conventional full‐field CBCT. The low‐dose full‐FOV scan provides complete anatomical information, whereas the conformal scan delivers higher fluence to projection paths contributing to the target region and reduces direct irradiation of non‐target regions outside the dynamic aperture. As a result, non‐target tissues may receive less direct exposure, while the target‐region dose distribution may be locally redistributed. Because patient‐specific organ dose, dose‐volume metrics, and Monte Carlo dose calculations were not performed in this study, the dose comparison should be interpreted qualitatively. Future work will quantify organ‐specific imaging dose with more patient data collected.

Although this study achieved promising results, several limitations remain. the RWLS reconstruction algorithm requires manual setting of several parameters, including regularization weights and the Huber parameter of TV regularization. These parameters strongly influence reconstruction results and are currently selected mainly through experience and trial and error. Different patients and anatomical sites may require different parameter settings, which increases the complexity of clinical implementation. Future studies should investigate automatic parameter optimization based on image‐quality metrics or deep learning models that predict patient‐ and site‐specific reconstruction parameters.

Compared with a conventional single‐rotation clinical CBCT workflow, the proposed dual‐scan protocol introduces an additional acquisition because it combines a low‐dose full‐FOV scan with a conformal scan. If the same gantry rotation speed and projection number are used, the acquisition time would therefore increase relative to standard CBCT. In clinical implementation, this time overhead could potentially be reduced by using fewer projections or fewer control points for the conformal scan, faster gantry rotation, or adaptive angular sampling. The reconstruction time is also expected to be longer than that of FDK because the proposed RWLS reconstruction is iterative, although the use of preconditioned conjugate‐gradient optimization and separable quadratic surrogates can accelerate convergence. Formal runtime benchmarking on a clinical reconstruction workstation was not performed in this proof‐of‐concept study and should be included in future work before clinical translation.

The hardware feasibility of dynamic collimation also needs to be considered. In the simulation studies, dynamic collimation was implemented using an MLC model, which provides more flexible target‐conformal field shaping. However, most currently available clinical kV‐CBCT systems are equipped with movable rectangular collimators rather than dedicated imaging MLCs. Therefore, the MLC‐based simulations should be interpreted as an evaluation of an advanced conformal‐collimation implementation, whereas the physical phantom experiment demonstrates a more immediately available implementation using the movable kV collimator of the Varian Edge OBI system. More irregular target conformity would require a dedicated kV imaging MLC or another fast motorized multi‐leaf/jaw system synchronized with gantry angle and kV projection acquisition. Such implementation would require careful calibration of leaf position, synchronization accuracy, transmission and penumbra characterization, mechanical clearance evaluation, and routine imaging quality assurance.

## 5 CONCLUSION

This study developed a patient‐specific dual‐scan conformal CBCT imaging method based on dynamic collimation, target‐region‐based angular intensity optimization, and regionally weighted iterative reconstruction. By dynamically shaping the imaging field to conform to the planned target region and allocating more photons to projections that contribute to that region, the proposed method improves local image quality while maintaining full‐FOV information through a low‐dose full‐field scan. In the digital phantom and patient‐data simulations, dynamic collimation was implemented with an MLC model, whereas in the measured anthropomorphic head phantom experiment it was implemented using the movable kV collimator of the Varian Edge OBI system. Across all evaluations, the proposed method consistently improved target‐region image quality compared with conventional FDK reconstruction, attenuation‐based TCM, and iterative reconstruction alone.

## AUTHOR CONTRIBUTIONS

**Yuxiang Liu**: Conceptualization; methodology; formal analysis; investigation; data curation; writing—original draft; visualization. **Siqi Yuan**: Methodology; data curation. **Xin feng**: Validation; formal analysis. **Jianrong Dai**: Conceptualization; methodology; software; investigation; writing—review & editing; visualization; funding acquisition; project administration. We confirm that all co‐authors contributed to the study.

## CONFLICT OF INTEREST STATEMENT

The authors declare no conflicts of interest.

## ETHICS STATEMENT

This retrospective study was approved by the review board of Cancer Hospital, Chinese Academy of Medical Sciences, and informed consent was waived.

## Supporting information




**Supporting Figure 1**: ROIs selected for CNR and SNR calculation(A) prostate cancer patient.(B) anthropomorphic head phantom.

**Supporting Table 1**: Comparison of acquisition and reconstruction schemes. **Supporting Table 2**: SSIM and RRMSE results for the patient‐data simulations.

## Data Availability

All supporting data are included as supplementary files with this manuscript. Additional raw data can be obtained from the corresponding author upon reasonable request.
