# Supplementary figures and images for: Dual‐scan conformal cone‐beam CT for targeted image‐quality improvement using dynamic collimation
Source: J Appl Clin Med Phys. 2026 Jun 23;27(7):e70671. doi: 10.1002/acm2.70671 (PMC13291214; doi:10.1002/acm2.70671)

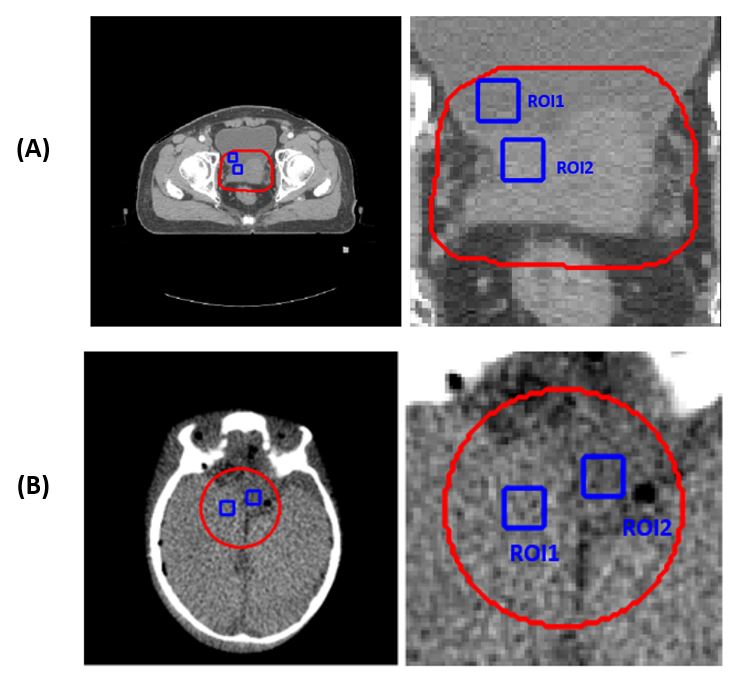

Supplement: Supplementary file 1 — Supporting Figure 1: ROIs selected for CNR and SNR calculation(A) prostate cancer patient.(B) anthropomorphic head phantom. [file ACM2-27-e70671-s001.jpg]
