# Supplementary material for: Dual‐scan conformal cone‐beam CT for targeted image‐quality improvement using dynamic collimation
Source: J Appl Clin Med Phys. 2026 Jun 23;27(7):e70671. doi: 10.1002/acm2.70671 (PMC13291214; doi:10.1002/acm2.70671)
Supplement: Supplementary file 2 — Supporting Table 1: Comparison of acquisition and reconstruction schemes. Supporting Table 2: SSIM and RRMSE results for the patient‐data simulations. [file ACM2-27-e70671-s002.docx]

Appendix

Table S1. Comparison of acquisition and reconstruction schemes.

| **Method abbreviation** | **Scanning mode** | **Beam intensity modulation** | **Reconstruction algorithm** |
| --- | --- | --- | --- |
| FDK-s1 | One full-field scan | Constant | FDK |
| FDK-TCM-s1 | One full-field scan | Attenuation-based TCM | FDK |
| IT-s1 | One full-field scan | Constant | RWLS |
| FDK-TCM-s2 | One low-dose full-field scan + one conformal scan | Attenuation-based TCM | FDK |
| IT-TCM-s2 | One low-dose full-field scan + one conformal scan | Attenuation-based TCM | RWLS |
| Proposed | One low-dose full-field scan + one conformal scan | Target-region-based intensity optimization | RWLS |

Table S2. SSIM and RRMSE results for the patient-data simulations.

| **Dataset** | **Method** | **SSIM** | **RRMSE** |
| --- | --- | --- | --- |
| NPC | FDK-s1 | 0.700 | 0.426 |
|  | FDK-TCM-s1 | 0.701 | 0.426 |
|  | IT-s1 | 0.812 | 0.426 |
|  | FDK-TCM-s2 | 0.737 | 0.426 |
|  | IT-TCM-s2 | 0.842 | 0.425 |
|  | Proposed | 0.846 | 0.424 |
| Prostate | FDK-s1 | 0.612 | 0.503 |
|  | FDK-TCM-s1 | 0.647 | 0.504 |
|  | IT-s1 | 0.729 | 0.502 |
|  | FDK-TCM-s2 | 0.760 | 0.510 |
|  | IT-TCM-s2 | 0.758 | 0.502 |
|  | Proposed | 0.761 | 0.501 |
